# Polyorchidism: A Rare Finding in Inguinal Swelling

**Published:** 2013-07-10

**Authors:** Dhiraj Parihar, Yogender Singh Kadian, Kamal Nain Rattan

**Affiliations:** Department of Surgery, B P S Government Medical College for Women Khanpur-Kalan; Department of Pediatric Surgery, Pt. B.D.Sharma PGIMS, Rohtak (Haryana); Department of Pediatric Surgery, Pt. B.D.Sharma PGIMS, Rohtak (Haryana)

**Dear Sir,**

Polyorchidism is a rare congenital anomaly with most common occurrence of three testes (triorchidism). Polyorchidism is more common on left side. To date, in children less than 50 cases are reported.[1,2] The majority of patients are asymptomatic or present with painless inguinoscrotal masses, undescended testis, and rarely, torsion of the supernumerary testis. In children the appropriate management of polyorchidism remains unclear. With the advances in imaging techniques the former practice of removing the supernumerary testes has changed. The conservative surveillance of polyorchidism in cases with a normal radiological appearance and negative tumor-markers is an accepted practice especially in the pediatric population.[3] We have encountered a child with right sided triorchidism who was managed by preservation of both the testes.

A 3-year-old boy was admitted with right sided inguinal swelling for 7 days. Parents had noticed that both the testis were descended into the scrotum since birth but the right sided scrotum was empty since the child had developed the inguinal swelling. On examination, he was in obvious discomfort. Local examination of inguinoscrotal area revealed a normally descended left testis and an irreducible swelling in the right inguinal area. The right scrotum was empty but it was well developed. Ultrasonography of the swelling revealed echogenic shadows consistent with intestines and testis. After the routine haematological investigations, patient was operated with a suspicion of an obstructed inguinal hernia. At operation, after reduction of hernia contents it was found that there were two testes on the right side which were drained by a common vas deferens (Fig. 1). Herniotomy was performed and both the testes were placed into the ipsilateral scrotum by creating subdartos pouch. The patient is well at follow up.

**Figure:**
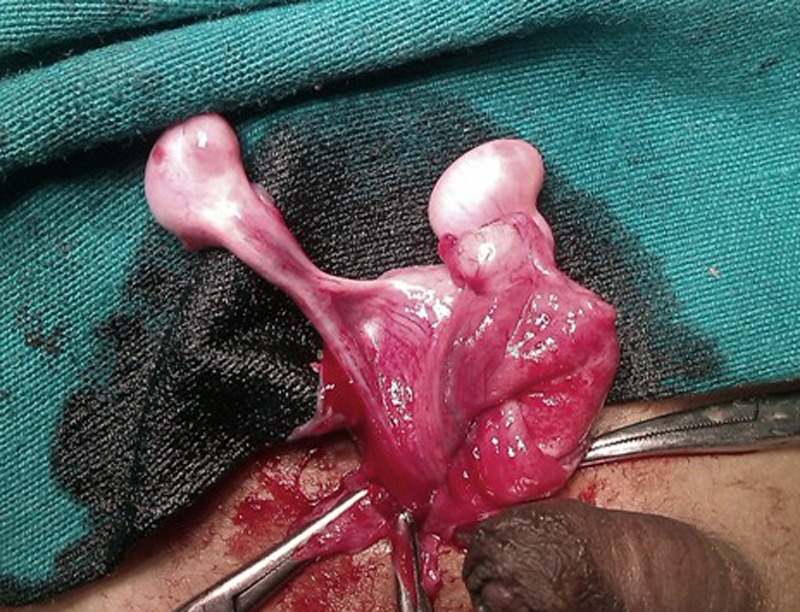
Figure 1: The operative photograph showing two testes on the right side in the inguinal area and draining via a common vas.

The majority of supernumerary testes are located in the scrotal region (66%) followed by inguinal (23%), and abdominal (9%) positions.[1] The exact etiology of polyorchidism is unknown, however, an anomalous appropriation of cells in the genital ridge before 8 weeks of gestation, duplication or transverse versus longitudinal division of the urogenital ridge, incomplete degeneration of mesonephrons and development of peritoneal bands are few theories about its genesis.[1,2]

Leung described the first anatomical classification which was based on the possible embryological variations: Type I: Supernumerary testis lacks an epididymis or vas deferens and has got no attachment to the usual testis (division of genital ridge only); Type II: The supernumerary testis drains into epididymis of usual testis and they share a common vas deferens (division of genital ridge occurs in the region where the primordial gonads are attached to the metanephric ducts, although the mesonephrons and metanephric ducts are not divided); Type III: The supernumerary testis has its own epididymis and both epididymis of ipsilateral testes drain into a common vas deferens (complete transverse division of genital ridge as well as mesonephrons); Type IV: Complete duplication of testis, epididymis and vas (vertical division of genital ridge and mesonephrons).[4] In this patient the right sided polyorchidism was of Type III.

The incidence of testicular malignancy in polyorchidism is between 5.7–7% and was found only in a non-scrotal (abdominal or inguinal) supernumerary testis. Management of polyorchidism has been subjected to much debate. With recent improvements in imaging techniques such as ultrasound and MRI, most cases of polyorchidism can be diagnosed and followed up accurately without any need for surgical exploration or histological examination.[3] However, if the patient is symptomatic or there is a suspicion for malignancy surgical exploration, biopsy and orchidectomy are recommended when the intraoperative frozen section is positive for malignancy.[5] But there is no consensus in the literature regarding the management of polyorchidism especially in situations like the present case, where the supernumerary testes is found incidentally while operating on a obstructed hernia or an undescended testis. In such a case the orchidectomy of the supernumerary testes is advocated only when it is atrophic, separated from the normal testis or without connection to the vas deferens.[6] Moreover it has been seen on imaging during follow up of such cases that accessory testes may have reproductive potential.[6] On the basis of the available literature and taking into the above mentioned criteria, it is appropriate to preserve the supernumerary testis by performing scrotal orchidopexy as also done in the present case.

## Footnotes

**Source of Support:** Nil

**Conflict of Interest:** None declared

